# Application of Apparent Metabolizable Energy versus Nitrogen-Corrected Apparent Metabolizable Energy in Poultry Feed Formulations: A Continuing Conundrum

**DOI:** 10.3390/ani11082174

**Published:** 2021-07-22

**Authors:** M. Reza Abdollahi, Markus Wiltafsky-Martin, Velmurugu Ravindran

**Affiliations:** 1 Monogastric Research Centre, School of Agriculture and Environment, Massey University, Palmerston North 4442, New Zealand; V.Ravindran@massey.ac.nz; 2 Evonik Operations GmbH, Animal Nutrition, Rodenbacher Chaussee 4, 63457 Hanau, Germany; Markus.wiltafsky-martin@evonik.com

**Keywords:** poultry, apparent metabolizable energy, nitrogen-corrected apparent metabolizable energy, nitrogen retention

## Abstract

**Simple Summary:**

Despite some limitations, the metabolizable energy system has been extensively used for describing the available energy in ingredients and for formulating complete poultry feeds. Three methods, namely direct, difference (substitution), and regression, or modifications thereof, have been employed to measure the apparent metabolizable energy (AME) of feeds and ingredients for poultry. The AME of feed ingredients are often corrected for zero nitrogen (N) retention to estimate the N-corrected AME (AMEn). Although the need for N-retention corrections has been intensely debated and challenged ever since the advent of the AME system, no definitive conclusion has been reached and the majority of poultry diets today are formulated to meet the requirements for AMEn rather than AME. There is limited information on the effect of zero N-retention correction on the energy value of major protein sources. The aim of this investigation was to understand the consequences of correction to zero N retention to the energy values of samples of several protein sources differing in protein quality. Based on the data presented herein, correcting AME values to zero N retention for modern fast-growing broilers penalizes the energy value of all major protein sources and is of higher magnitude for ingredients with higher protein quality.

**Abstract:**

In the present investigation, N retention, AME, and AMEn data from six energy evaluation assays, involving four protein sources (soybean meal, full-fat soybean, rapeseed meal and maize distiller’s dried grains with solubles [DDGS]), are reported. The correction for zero N retention, reduced the AME value of soybean meal samples from different origins from 9.9 to 17.8% with increasing N retention. The magnitude of AME penalization in full-fat soybean samples, imposed by zero N correction, increased from 1.90 to 9.64% with increasing N retention. The Δ AME (AME minus AMEn) in rapeseed meal samples increased from 0.70 to 1.09 MJ/kg as N-retention increased. In maize DDGS samples, the correction for zero N retention increased the magnitude of AME penalization from 5.44 to 8.21% with increasing N retention. For all protein sources, positive correlations (*p* < 0.001; r = 0.831 to 0.991) were observed between the N retention and Δ AME. The present data confirms that correcting AME values to zero N retention for modern broilers penalizes the energy value of protein sources and is of higher magnitude for ingredients with higher protein quality. Feed formulation based on uncorrected AME values could benefit least cost broiler feed formulations and merits further investigation.

## 1. Introduction

Amongst the broad spectrum of factors affecting the efficiency of production, an adequate and balanced supply of energy and macro- and micro-nutrients is of the greatest importance. Energy is not a nutrient, but a property of energy-yielding nutrients such as carbohydrates, lipids, and protein. Dietary energy represents the major quantitative and costliest component in poultry feed formulations, and is the first component to be considered when the diets are being balanced. Dietary energy also controls the feed consumption, which is the major driver of bird growth. Therefore, an accurate evaluation of the available energy content of ingredients is important for precise and cost-effective feed formulations.

Since its introduction in the mid-1950′s [1], the metabolizable energy (ME) system has been commonly accepted and extensively used for describing bird’s energy requirements and available energy of individual feed ingredients, and for formulating complete poultry feeds. The ME system is not a perfect energy system, as it presents several limitations [2,3,4]. The measurement of ME is based on excreta, meaning that it contains energy originated from dietary energy as well as endogenous metabolic and urinary energy which is of non-dietary origin. It is also voided along with feces and includes the energy loss or gain from caecal fermentation and microbial mass. However, its simplicity, ease of measurements, lack of need for bird euthanasia, and the fact that it accounts for most of the energy losses after digestion and metabolism have positioned the ME as the globally accepted system for describing the available energy in ingredients for poultry.

In the ME system, the available energy is calculated as the difference between the dietary gross energy (GE) ingested and the GE excreted in the excreta (feces and urine) [1,5,6], and is referred to as apparent ME (AME).

AME (MJ/kg diet) = [(Feed intake × GE_diet_) − (Excreta output × GE_excreta_)]/Feed intake.

Three methods, namely direct, difference (substitution) and regression method, or modifications thereof, have been employed to measure the AME of feeds and ingredients for poultry. Each method has its own merits and drawbacks, and differs in the way the assay diets are prepared. Within each method, the excreta collection can be performed as either by total collection, which is the most preferred method, or partial excreta collection (marker method) using the ratio of an indigestible marker present in diet and excreta. The direct method is the most widely used method to estimate AME, mainly because of the simplicity of the assay diet and calculations. In this method, which is applied mainly for cereal grains, only one ingredient is used as the sole source of energy in the test diet [7]. A limitation of this method is that it cannot be used for poorly palatable ingredients. Furthermore, because cereals are nutritionally imbalanced, these diets will have adverse effects on the body functions when fed for several days and cannot be used for longer feeding periods.

The difference or substitution method is another assay that is used to evaluate the AME of ingredients with poor palatability, high protein content, or high levels of anti-nutritional factors. This method requires the formulation of two sets of diets, a basal (reference diet) and the assay diet. The basal diet consists of a mixture of ingredients, typically a maize-soybean meal diet, whereas the assay diet is developed by replacing a portion of the basal diet with the test ingredient. Depending on the ingredient, different substitution levels of the basal diet by the test ingredient are considered [8,9]. Although this method is a good alternative to the direct method as the reference diet is a standard diet, the difference method has some disadvantages as the AME of the test ingredient can be influenced by the composition of the basal diet and the accuracy of basal diet AME determination. Though the difference method assumes that there is no interaction between the basal diet and the test ingredients [10], this assumption is not always correct. Moreover, the inclusion level of the test ingredient will affect the AME value [11].

The third method of estimating the AME of ingredients is the regression method. In this method, a basal diet and test diets, in which the basal diet is replaced by at least two levels of the ingredient, are fed. The energy value of individual diets is compared to the corresponding inclusion level of the ingredient and extrapolation of energy to the equivalency of 100% inclusion predicts the AME of the test ingredient [12].

Once determined, the AME is often corrected for zero nitrogen (N) retention, and the resultant estimate is referred to as the N-corrected AME (AMEn). The assumption for zero N correction is that the oxidation of protein retained as body tissue will yield uric acid that has a GE per gram of N [4]. Two correction factors are commonly used for N correction: the GE value of uric acid, which is 0.034 MJ/kg [1], or the GE value of N-containing compounds found in chicken urine, which is 0.037 MJ/kg [13].

AMEn_diet_ (MJ/kg) = AME_diet_ − [N retention (g/kg) × Correction factor/1000]

Although the ME system is generally used in the poultry industry for describing the energy requirements of birds and formulating the diets, a practical issue faced by commercial nutritionists is the conundrum of whether to use the AME or the AMEn in poultry feed formulations. This issue has been intensely debated ever since the advent of the ME system [4,14,15], with no definitive conclusion. Lopez and Leeson [4], using a series of commercial diets for broilers, reported that the correction for zero N retention penalized the dietary AME content from 3.8% (at d 49) to 5.3% (at d 7). Lopez and Leeson [15] reported an AMEn:AME ratio of 0.95–0.99 for maize, implying that N correction imposed a penalty of 1.0 to 5.0% in the AME of maize. For soybean meal (SBM), the AMEn:AME ratio was 0.88–0.93, suggesting a higher penalty from 7.0 to 12.0%. A similar trend of greater penalization could also be expected for ingredients that have been properly heat-treated, and have higher protein quality and N retention, compared to those under- or over-processed. The implication of this discussion may be that the application of AMEn values will underestimate the true energy contribution, to dietary energy, of ingredients with high protein content or/and high-quality. In the investigation presented herein, unpublished data from AME assays in our laboratory were analyzed to better understand the consequences of correction to zero N retention to the energy values of samples of several protein sources differing in protein quality. For the purpose of current analysis, N retention is considered as a good indicator of protein quality [16].

## 2. Materials and Methods

The N retention, AME, and AMEn data from six energy evaluation assays, involving four protein sources, are reported herein: 28 SBM samples from different origins (assay 1), 16 full-fat soybean (FFSB) samples exposed to different wet heating and autoclaving conditions (assay 2), 9 SBM samples exposed to different autoclaving conditions (assay 3), 9 rapeseed meal (RSM) samples exposed to different autoclaving conditions (assay 4), 9 maize distiller’s dried grains with solubles (DDGS) samples exposed to different autoclaving conditions (assay 5), and 10 FFSB samples exposed to different autoclaving conditions (assay 6). For the sake of completion and comparison, published data for cereals (maize, wheat, sorghum, and barley) generated in our laboratory [17,18] are also considered.

For protein sources, the AME was determined by the difference method. In this method, a maize-soybean basal diet was formulated [19], and the test diets, each containing different protein sources, were developed by replacing (*w*/*w*) 300 g/kg of the basal diet with the protein source. The AME of cereal grains was determined using the direct method. The assay diets contained 962 g/kg of the test cereal as the only source of energy in the diet.

All experimental procedures were approved by the Massey University Animal Ethics Committee. One-day-old male broilers (Ross 308), obtained from a commercial hatchery, were raised in floor pens (stocking density of maximum 25 kg/m^2^), and fed a commercial broiler starter diet (230 g/kg CP) until day 21. Feed and water were available at all times. The temperature was maintained at 32°C during the first week and gradually decreased to approximately 23 °C by the end of the third week. On day 21, birds of uniform body weight were selected and randomly assigned to experimental cages (6 birds per cage; space allocation of 600 cm^2^ per bird), and 4 (assay 1) or 6 (all other assays) replicate cages were randomly assigned to each of the assay diet. The floor pens and experimental cages were housed in an environmentally controlled poultry house supplying 20 h of fluorescent illumination per day. The experimental design in all the assays was a completely randomized design, and cage means served as the experimental unit. The assays were conducted by the classical total excreta collection method. The diets, in mash form, were fed for 7 days, with the first 3 days serving as an adaptation period. During the last 4 days, feed intake was monitored, and the excreta were collected daily, weighed, and pooled within a cage. Pooled excreta were mixed well, and representative samples were obtained and freeze-dried. Dried excreta samples were ground to pass through a 0.5-mm sieve and stored in airtight plastic containers at 4 °C until laboratory analyses. The dry matter, gross energy, and N of the diet and excreta samples were determined. Dry matter was determined using standard procedures (Methods 930.15) [20]. Gross energy was determined by adiabatic bomb calorimeter (Gallenkamp Autobomb, London, UK) standardized with benzoic acid. Nitrogen was determined by combustion (Method 968.06) [20] using a carbon nanosphere-200 carbon, N and sulfur auto-analyzer (rapid MAX N exceed, Elementar Analysensysteme GmbH, Donaustraße, Hanau, Germany).

The AME value of protein sources were calculated using the following formulas:

AME_diet_ (MJ/kg) = [(Feed intake × GE_diet_) − (Excreta output × GE_excreta_)]/Feed intake

AME_protein source_ (MJ/kg) = [AME of test diet − (AME of basal diet × 0.70)]/0.30

The AME value of cereals were calculated using the following formulas:

AME_diet_ (MJ/kg) = [(Feed intake × GE_diet_) − (Excreta output × GE_excreta_)]/Feed intake

AME_grain_ (MJ/kg) = AME of test grain diet × (100/96.2)

For all studies, N retention, as a percentage of N intake, was determined as follows:

N retention (%) = 100 × [((Feed intake × N_diet_) − (Excreta output × N_excreta_))/(Feed intake × N_diet_)]

The correction for zero N retention was made using a factor of 36.54 kJ per gram N retained in the body, as described by Titus et al. [13].

## 3. Results

The N retention, AME, and AMEn of the SBM samples from different origins in assay 1 ranged from 43.6 to 51.7% (average of 46.5%), 6.63 to 11.54 MJ/kg (average of 9.32 MJ/kg), and from 5.48 to 10.10 MJ/kg (average of 8.07 MJ/kg), respectively (Table 1).

**Figure 1:**
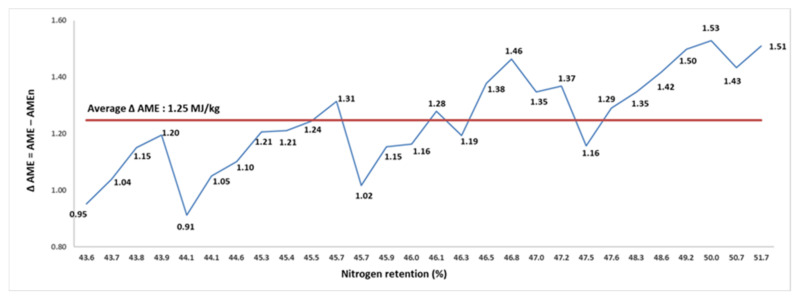
The relationship (r = 0.831; *p* < 0.001) between nitrogen retention (% intake) and the difference (Δ) between apparent metabolizable energy (AME; MJ/kg dry matter) and nitrogen-corrected AME (AMEn; MJ/kg dry matter) in 28 soybean meal samples for broilers (assay 1).

There was an average of 1.25 MJ/kg difference between AME and AMEn, and the difference increased from 0.91 MJ/kg in the sample with 44.1% N retention to 1.53 MJ/kg in the sample with 50.0% N retention. The correction for zero N retention imposed an average reduction of 13.5% in the AME value of SBM samples. However, the percentage of penalization varied from 9.9 to 17.8%, and increased with increasing N retention. The N retention and the difference (Δ AME) between AME and AMEn of SBM from different origins were positively correlated (*p* < 0.001; r = 0.831), as exemplified in Figure 1. Interestingly, these findings demonstrate that N correction imposes a greater penalty on the available energy of SBM samples with higher protein quality.

The findings for N retention, AME, and AMEn of 16 FFSB samples from assay 2 for broilers are shown in Table 2. Different samples of FFSB were manufactured from a single batch of raw FFSB by exposing to different processing (wet heating and autoclaving) conditions.

**Figure 2:**
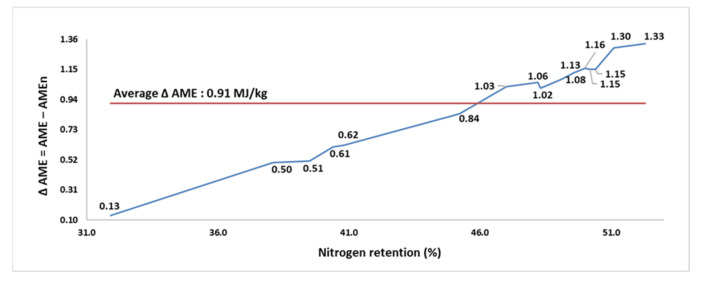
The relationship (r = 0.995; *p* < 0.001) between nitrogen retention (% intake) and the difference (Δ) between apparent metabolizable energy (AME; MJ/kg dry matter) and nitrogen-corrected AME (AMEn; MJ/kg dry matter) in 16 full-fat soybean samples for broilers (assay 2).

The processing condition had a substantial influence on N retention, resulting in a wide range from 31.9 to 52.3% (average of 45.8%). The AME and AMEn of the FFSB samples ranged from 6.84 to 14.84 MJ/kg (average of 13.31 MJ/kg), and from 6.71 to 13.61 MJ/kg (average of 12.39 MJ/kg), respectively. The Δ AME increased from 0.13 MJ/kg in the sample with the lowest N retention to 1.33 MJ/kg in the sample with the highest N retention. The magnitude of AME penalization, imposed by zero N correction, varied from 1.90 to 9.64% and increased with increasing N retention of the FFSB samples. For every 10 percentage points increase in N retention above 31.9%, the correction for zero N retention penalized the AME value of FFSB samples by 0.59 MJ/kg. A strong and positive correlation (*p* < 0.001; r = 0.995; Figure 2) existed between the N retention and the Δ AME.

**Figure 3:**
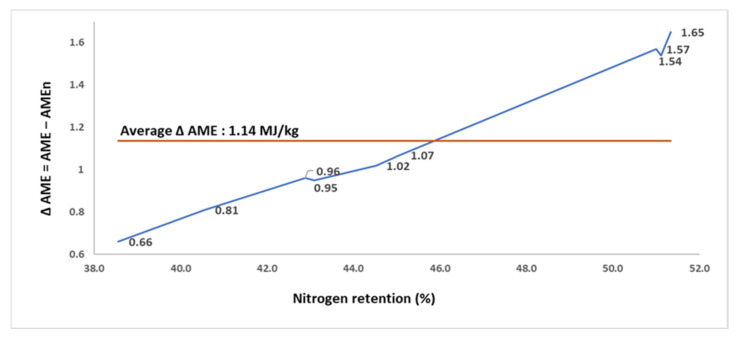
The relationship (r = 0.994; *p* < 0.001) between nitrogen retention (% intake) and the difference (Δ) between apparent metabolizable energy (AME; MJ/kg dry matter) and nitrogen-corrected AME (AMEn; MJ/kg dry matter) in 9 soybean meal samples for broilers (assay 3).

The N retention, AME, and AMEn of 9 SBM samples, developed from a single batch of SBM but exposed to different autoclaving conditions (assay 3) are summarized in Table 3. The N retention, AME, and AMEn of the SBM samples ranged from 38.6 to 51.3% (average of 45.4%), 10.0 to 12.18 MJ/kg (average of 11.10 MJ/kg), and from 9.34 to 10.61 MJ/kg (average of 9.96 MJ/kg), respectively. The Δ AME (with an average of 1.14 MJ/kg) increased from 0.66 MJ/kg in the sample with lowest N retention to 1.65 MJ/kg in the sample with the greatest N retention, and was strongly correlated (*p* < 0.001; r = 0.994; Figure 3) with the N retention. When the AME values were corrected for zero N retention, a 12.7 percentage points increase in N retention (51.3 vs. 38.6%) increased the Δ AME by 0.99 MJ/kg, an increase in 2.5 folds.

**Figure 4:**
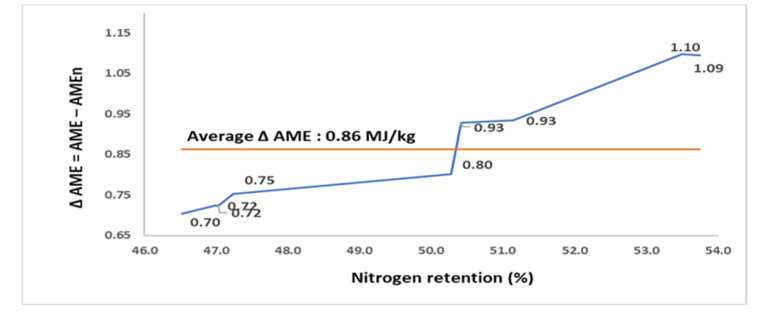
The relationship (r = 0.972; *p* < 0.001) between nitrogen retention (% intake) and the difference (Δ) between apparent metabolizable energy (AME; MJ/kg dry matter) and nitrogen-corrected AME (AMEn; MJ/kg dry matter) in 9 rapeseed meal samples for broilers (assay 4).

The N retention AME, and AMEn of 9 RSM samples, sourced from a single batch of raw RSM but which underwent different autoclaving conditions (assay 4), are compared in Table 4. The autoclaving conditions resulted in RSM samples being varied in N retention from 46.5 to 53.7%, AME from 7.71 to 8.94 MJ/kg, and AMEn from 6.99 to 7.84 MJ/kg. The N retention and Δ AME of RSM samples were positively correlated (*p* < 0.001; r = 0.972; Figure 4). The Δ AME increased from 0.70 MJ/kg in the sample with the poorest N retention to 1.09 MJ/kg in the sample with the greatest N retention.

**Figure 5:**
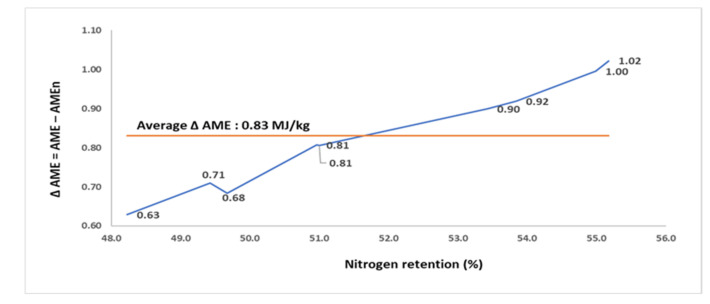
The relationship (r = 0.991; *p* < 0.001) between nitrogen retention (% intake) and the difference (Δ) between apparent metabolizable energy (AME; MJ/kg dry matter) and nitrogen-corrected AME (AMEn; MJ/kg dry matter) in 9 maize DDGS samples for broilers (assay 5).

The N retention, AME, and AMEn of the 9 maize DDGS samples (assay 5) are summarized in Table 5. A single batch of a maize DDGS was exposed to different autoclaving conditions to develop the 9 samples. The N retention of the nine samples varied from 48.2 to 55.2% with an average of 51.9%. The AME and AMEn ranged from 11.56 to 12.58 MJ/kg (average of 12.16 MJ/kg), and from 10.93 to 11.58 MJ/kg (average of 11.33 MJ/kg), respectively. The correction for zero N retention reduced the AME by an average of 6.81%, with the magnitude of AME penalization increasing from 5.44% in the sample with the lowest N retention to 8.21% in the sample with the highest N retention. A positive correlation (*p* < 0.001; r = 0.991; Figure 5) was observed between the percentage of N retention and the Δ AME.

**Figure 6:**
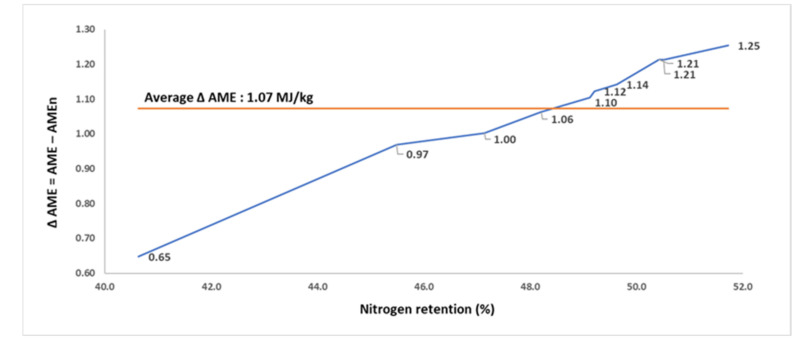
The relationship (r = 0.994; *p* < 0.001) between nitrogen retention (% intake) and the difference (Δ) between apparent metabolizable energy (AME; MJ/kg dry matter) and nitrogen-corrected AME (AMEn; MJ/kg dry matter) in 10 full-fat soybean samples for broilers (assay 6).

The findings for N retention, AME, and AMEn of the 10 FFSB samples, from a study in 2019, for broilers are summarized in Table 6. The FFSB samples were manufactured from a single batch of FFSB exposed to different autoclaving conditions. The N retention of FFSB samples differed from 40.6 to 51.7%, AME from 11.05 to 15.54 MJ/kg, and AMEn from 10.41 to 14.29 MJ/kg. The Δ AME increased from 0.65 MJ/kg in the sample with the poorest N retention to 1.25 MJ/kg in the sample with the highest N retention. The N retention and Δ AME of FFSB samples were positively correlated (*p* < 0.001; r = 0.994; Figure 6).

## 4. Discussion

The purpose of the current exercise is to address the conundrum of whether to use the AME or AMEn in poultry feed formulations. Interestingly, industry nutritionists are often unaware of the differences between the two estimates, or which one is being used in their formulation matrices. The correction for zero N retention was initially introduced to convert the AME values to N equilibrium and to eliminate the variation associated with the amount of N that is deposited as protein tissue and not oxidized in the body to provide energy [15]. The major justification for this correction was that energy evaluation assays, such as AME, diets, and feed ingredients, should be evaluated only for their contribution to energy supply and not promoting N retention [14]. The original intention for introducing the correction for zero N retention was to eliminate the differences in N retention caused by experimental factors, including dietary treatments, feed additives, ingredients, age of birds and bird strains, evaluated in the same AME assay, but not across assays [4]. It follows that the AMEn values generated in different AME assays are not comparable and should not be considered together, for example as in feed formulations [21,22,23,24].

The need for N retention correction, however, has been questioned by some researchers. Lopez and Leeson [4] opined that, with current specialization in poultry nutrition, the species comparison for energy evaluation is not relevant anymore, and the correction for zero N retention is unnecessary. The concept of N correction for broilers has also been challenged because modern broiler strains have comparable protein accretion, and therefore, comparisons are not critical [15]. Moreover, the test diets used in ME assays are not representative of commercial diets and not balanced for most of the nutrients (relative to their requirements), especially amino acids. The use of such imbalanced diets not only reduces the protein accretion compared to birds fed balanced diets under commercial situations, but also lowers birds’ ability to derive energy from these diets. Unbalanced amino acid assay diets increase N excretion, underestimating the energy value of ingredients due to the energetic cost related to uric acid synthesis [15,25]. The correction of AME values to zero N retention will further penalize the energy content of feed ingredients, especially those with high-protein contents.

The present analyses show that correcting the AME to zero N retention heavily penalizes the energy value of high-protein ingredients, such as SBM (average of 1.25 MJ/kg; Table 1), over low-protein ingredients, such as cereal grains (average of 0.30 MJ/kg; Table 7), by almost four-fold. This difference is due to the associated higher protein accretion with high-protein ingredients and correction for zero N correspondingly reduces their true energy value [15,26]. Even within SBM samples (Table 1), the correction for zero N retention could harshly penalize the samples with highest protein quality and retention. In the data reported in Table 1, the ME content of the SBM sample with the highest N retention of 51.7% was penalized 0.56 MJ/kg (1.51 vs. 0.95 MJ/kg) more than that with the lowest N retention of 43.6%. From a practical point of view, using N-corrected AME values for high-protein ingredients, such as SBM, in feed formulation could underestimate the energy value of SBM originating from countries that manufacture quality SBM.

Similar concerns exist for properly heat-treated protein ingredients with higher protein quality, compared to the same ingredient that was either under- or over-processed. As the same principle is applied in the N correction of AME values, regardless of ingredient type or the extent of processing, ingredients that have undergone proper thermal process and have higher protein quality are the ones that are heavily penalized for their ME content. The findings reported in Table 2 Table 3 Table 4 Table 5 and Table 6 provide further support to the above conclusion. The N correction of AME values could penalize ME content of properly processed FFSB by 1.20 MJ/kg (1.33 vs. 0.13 MJ/kg; Table 2), SBM by 0.99 MJ/kg (1.65 vs. 0.66 MJ/kg; Table 3), RSM by 0.39 MJ/kg (1.09 vs. 0.70 MJ/kg; Table 4), and maize DDGS by 0.39 MJ/kg (1.02 vs. 0.63 MJ/kg; Table 5) more than their corresponding samples that were under- or over-processed. The use of AMEn values by poultry nutritionists, as part of a decision-making tool for selecting ingredients for practical feed formulations, could be misleading when choosing among different quality samples of the same ingredient.

Since the same correction factor is used for all ingredients to convert the AME to AMEn, the N correction can also penalize the energy value of cereal grains. Table 7 summarizes recent data on the N retention, AME, and AMEn of major cereals from our laboratory [17,18]. The penalty imposed by the N correction to the AME ranged from 1.69% in maize to 2.46% in barley and was much lower compared to protein ingredients. The lower AME penalization in cereals compared to protein sources is expected on the basis of their lower protein content and N retention. It should also be noted that cereal inclusion in poultry diets is normally 2–3 times higher than that of protein sources, and therefore, the use of AMEn values for cereals in feed formulations could have a marked impact on dietary energy content, feed cost, and economic returns.

Under commercial conditions, poultry feed companies generally use tabulated energy values and/or prediction equations to estimate the energy content of ingredients and diets [2]. Most of the table energy values for poultry feed ingredients are based on AMEn [6,27,28,29]. In recent years, an array of predictive equations has been developed for the estimation of the ME of ingredients or compound feeds, from their chemical composition obtained by wet chemistry or near-infrared reflectance (NIRS) technology, for poultry [27,30,31,32,33,34,35,36]. Similar to tabulated energy values, the majority of ME values derived from predictive equations for poultry feed ingredients are also based on AMEn. Recently, based on an artificial neural network model, a mobile app, called AMEn Predictor [37], has been developed to predict AMEn values of ingredients commonly used in feed formulations for broilers, and seemingly used in several countries (Algeria, Brazil, Iran, Italy, UK, and USA). Moreover, energy requirements of poultry in NRC [6] and most strain recommendation manuals [38,39,40] are estimated and reported as N-corrected AME. Consequently, the majority of the poultry diets are being formulated to meet the requirements for AMEn rather than AME. From the forgoing discussion, it is clear that formulating to AMEn requirements could potentially impose unnecessary extra cost to the meat chicken industry, as modern broilers have been selected for maximum retention of nutrients, especially N and amino acids, and a high protein deposition is, therefore, a desired biological norm [4,15]. This aspect becomes even more pertinent with the current practice of formulating diets based on ideal protein concepts in modern fast-growing broilers, in which a high proportion of the ingested protein is accredited as muscle and not oxidized or stored as fat [2].

Table 8 presents examples of starter, grower, and finisher maize-soybean meal-based diets formulated using AME or AMEn values for maize and SBM. The AME and AMEn values used for formulation were 14.14 and 13.90 MJ/kg for maize, and 11.10 and 9.96 MJ/kg for SBM (average of 9 SBM samples from Table 3), respectively. Formulation to AME of maize and SBM, rather than AMEn, reduced the inclusion of soybean oil by 27.1, 25.0 and 22.9 g/kg, and feed cost by NZ$70, 64 and 59 per tonne in the starter, grower and finisher diets, respectively. There are at least two avenues by which the chicken meat industry could benefit from formulating diets to AME: (i) potential savings in feed costs and, therefore, improved production economics, especially with the ever-increasing global price of fat sources, and (ii) enhancing the physical quality of pellets by reduced dietary inclusion of fat [41,42]. However, this will require further applied research that considers broiler performance, market weight, and economic return.

Lopez and Leeson [15], using the determined AME or AMEn values for maize and SBM, formulated isoenergetic broiler starter (12.97 MJ/kg) and grower diets (13.60 MJ/kg), and reported that broilers fed diets, formulated using either AME or AMEn values for maize and SBM, performed remarkably equal, with a similar growth rate and comparable feed efficiency at 42 days of age. However, feed formulation using AME contents of maize and SBM reduced the fat supplementation in starter and grower diets by 22.7 and 24.8 g/kg, and resulted in less feed cost by US$10/ton compared to formulation based on AMEn contents. It was suggested that, within the scope of commercial broiler production, the AME better describes the available energy from cereals and protein sources, and feed formulation to AME values would benefit the production economy. It was also suggested that the energy value of SBM is underestimated due to the N correction. Based on data reported in the current study, it is clear that the undervaluation of energy due to the N correction is also relevant for all protein sources used in poultry diets and of higher magnitude for ingredients with better protein quality.

## 5. Conclusions

Energy is the major quantitative and costliest component in poultry diets and, therefore, the first step in feed evaluation is the assessment of available energy. Accuracy of metabolizable energy estimates is critical and imprecise AME matrices can increase feed cost and, through its effect on feed intake, can increase nutrient excretion into the environment. The data in the present study confirms that correcting AME values to zero N retention for modern fast-growing broilers penalizes the energy value of all feed ingredients that contribute to body protein deposition. The magnitude of decline in energy value due to zero N correction varies depending on the protein content and quality of the ingredient, imposing a heavy penalty to the protein sources with a higher protein quality. Therefore, using AMEn rather than AME values can be misleading for nutritionists when choosing feed ingredients for poultry feed formulation, resulting in the undervaluation and rejection of high-quality protein sources, and can consequently disadvantage production economics. Feed formulation to uncorrected AME values could possibly benefit the meat chicken industry, which merits more investigation.

## Figures and Tables

**Table 1:** The relationship between nitrogen (N) retention (% intake) and the difference (Δ) between apparent metabolizable energy (AME; MJ/kg dry matter) and N-corrected AME (AMEn; MJ/kg dry matter) in 28 soybean meal samples for broilers (assay 1) ^1.^.

Sample	N Retention ^2^	AME	AMEn	Δ AME	Δ AME as % of AME
1	43.6	9.06	8.11	0.95	10.5
2	43.7	8.64	7.60	1.04	12.0
3	43.8	8.42	7.27	1.15	13.7
4	43.9	8.77	7.57	1.20	13.6
5	44.1	7.63	6.71	0.91	12.0
6	44.1	10.60	9.55	1.05	9.91
7	44.6	9.09	7.99	1.10	12.1
8	45.3	9.47	8.27	1.21	12.7
9	45.4	9.40	8.19	1.21	12.9
10	45.5	9.11	7.86	1.24	13.7
11	45.7	9.21	7.90	1.31	14.3
12	45.7	8.66	7.64	1.02	11.7
13	45.9	6.63	5.48	1.15	17.4
14	46.0	10.44	9.28	1.16	11.1
15	46.1	9.27	7.99	1.28	13.8
16	46.3	8.91	7.72	1.19	13.4
17	46.5	9.78	8.40	1.38	14.1
18	46.8	8.20	6.74	1.46	17.8
19	47.0	9.19	7.84	1.35	14.7
20	47.2	9.46	8.09	1.37	14.5
21	47.5	10.07	8.91	1.16	11.5
22	47.6	10.30	9.01	1.29	12.5
23	48.3	9.03	7.69	1.35	14.9
24	48.6	9.27	7.86	1.42	15.3
25	49.2	10.27	8.77	1.50	14.6
26	50.0	10.20	8.67	1.53	15.0
27	50.7	11.54	10.10	1.43	12.4
28	51.7	10.33	8.82	1.51	14.6
Average	46.5	9.32	8.07	1.25	13.5

^1^ The samples were of different origins (United States, Brazil, Argentina, and India); Δ AME = AME − AMEn. ^2^ Data are sorted from lowest to the highest N retention.

**Table 2:** The relationship between nitrogen (N) retention (% intake) and the difference (Δ) between apparent metabolizable energy (AME; MJ/kg dry matter) and N-corrected AME (AMEn; MJ/kg dry matter) in 16 full-fat soybean samples for broilers (assay 2) ^1^.

Sample	N Retention ^2^	AME	AMEn	Δ AME	Δ AME as % of AME
1	31.9	6.84	6.71	0.13	1.90
2	38.1	12.19	11.69	0.50	4.10
3	39.5	13.45	12.94	0.51	3.79
4	40.4	13.20	12.59	0.61	4.62
5	40.8	11.71	11.09	0.62	5.29
6	45.2	13.83	12.99	0.84	6.07
7	47.0	14.05	13.02	1.03	7.33
8	48.2	13.26	12.20	1.06	7.99
9	48.3	13.23	12.21	1.02	7.71
10	49.1	14.54	13.46	1.08	7.43
11	49.6	14.74	13.61	1.13	7.67
12	50.0	14.43	13.27	1.16	8.04
13	50.2	14.73	13.58	1.15	7.81
14	50.4	14.10	12.95	1.15	8.16
15	51.1	14.84	13.54	1.30	8.76
16	52.3	13.79	12.46	1.33	9.64
Average	45.8	13.31	12.39	0.92	6.64

^1^ Different samples of full-fat soybean were manufactured from a single batch of raw full-fat soybean exposed to different processing (wet heating and autoclaving) conditions. ^2^ Data are sorted from lowest to the highest N retention. Δ AME = AME − AMEn.

**Table 3:** The relationship between nitrogen (N) retention (% intake) and the difference (Δ) between apparent metabolizable energy (AME; MJ/kg dry matter) and N-corrected AME (AMEn; MJ/kg dry matter) in 9 soybean meal samples for broilers (assay 3) ^1^.

Sample	N Retention ^2^	AME	AMEn	Δ AME	Δ AME as % of AME
1	38.6	10.00	9.34	0.66	6.60
2	40.6	10.58	9.77	0.81	7.66
3	42.9	11.00	10.04	0.96	8.73
4	43.1	10.89	9.94	0.95	8.72
5	44.5	11.13	10.11	1.02	9.16
6	45.1	10.61	9.54	1.07	10.08
7	51.0	12.18	10.61	1.57	12.89
8	51.1	12.03	10.49	1.54	12.80
9	51.3	11.45	9.80	1.65	14.41
Average	45.4	11.10	9.96	1.14	10.12

^1^ Different samples of soybean meal were manufactured from a single batch of conventional soybean meal exposed to different autoclaving conditions. ^2^ Data are sorted from lowest to the highest N retention. Δ AME = AME − AMEn.

**Table 4:** The relationship between nitrogen (N) retention (% intake) and the difference (Δ) between apparent metabolizable energy (AME; MJ/kg dry matter) and N-corrected AME (AMEn; MJ/kg dry matter) in 9 rapeseed meal samples for broilers (assay 4) ^1^.

Sample	N Retention ^2^	AME	AMEn	Δ AME	Δ AME as % of AME
1	46.5	8.16	7.45	0.70	8.63
2	47.0	7.71	6.99	0.72	9.39
3	47.0	8.22	7.50	0.72	8.80
4	47.2	8.06	7.30	0.75	9.34
5	50.3	8.08	7.28	0.80	9.91
6	50.4	8.21	7.28	0.93	11.30
7	51.1	8.44	7.51	0.93	11.07
8	53.5	8.94	7.84	1.10	12.29
9	53.7	8.75	7.66	1.09	12.51
Average	49.6	8.29	7.42	0.86	10.36

^1^ Different samples of rapeseed meal were manufactured from a single batch of raw rapeseed meal exposed to different autoclaving conditions. ^2^ Data are sorted from lowest to the highest N retention. Δ AME = AME − AMEn.

**Table 5:** The relationship between nitrogen (N) retention (% intake) and the difference (Δ) between apparent metabolizable energy (AME; MJ/kg dry matter) and N-corrected AME (AMEn; MJ/kg dry matter) in 9 maize distiller’s dried grains with solubles (DDGS) samples for broilers (assay 5) ^1^.

Sample	N Retention ^2^	AME	AMEn	Δ AME	Δ AME as % of AME
1	48.2	11.56	10.93	0.63	5.44
2	49.4	12.06	11.35	0.71	5.88
3	49.7	11.78	11.10	0.68	5.80
4	51.0	12.50	11.69	0.81	6.46
5	51.0	11.85	11.05	0.81	6.80
6	53.4	12.40	11.50	0.90	7.25
7	53.8	12.25	11.33	0.92	7.50
8	55.0	12.58	11.58	1.00	7.91
9	55.2	12.45	11.43	1.02	8.21
Average	51.9	12.16	11.33	0.83	6.81

^1^ Different samples of maize DDGS were manufactured from a single batch of commercial maize DDGS exposed to different autoclaving conditions. ^2^ Data were sorted from lowest to the highest N retention. Δ AME = AME − AMEn.

**Table 6:** The relationship between nitrogen (N) retention (% intake) and the difference (Δ) between apparent metabolizable energy (AME; MJ/kg dry matter) and N-corrected AME (AMEn; MJ/kg dry matter) in 10 full-fat soybean samples for broilers (assay 6) ^1^.

Sample	N Retention ^2^	AME	AMEn	Δ AME	Δ AME as % of AME
1	40.6	11.05	10.41	0.65	5.86
2	45.5	13.94	12.97	0.97	6.95
3	47.1	13.79	12.79	1.00	7.27
4	48.2	15.15	14.09	1.06	7.01
5	49.1	14.86	13.75	1.10	7.43
6	49.2	14.74	13.62	1.12	7.61
7	49.6	15.18	14.04	1.14	7.52
8	50.4	15.15	13.94	1.21	8.01
9	50.5	15.31	14.10	1.21	7.92
10	51.7	15.54	14.29	1.25	8.06
Average	48.2	14.47	13.40	1.07	7.36

^1^ Different samples of full-fat soybean were manufactured from a single batch of full-fat soybean exposed to different autoclaving conditions. ^2^ Data were sorted from lowest to the highest N retention. Δ AME = AME − AMEn.

**Table 7:** The nitrogen (N) retention (%), apparent metabolizable energy (AME; MJ/kg dry matter), N-corrected AME (AMEn; MJ/kg dry matter) and the difference (Δ AME) between AME and AMEn in four cereal grains for broilers.

	N Retention	AME	AMEn	Δ AME	Δ AME as % of AME	Reference
Maize	53.0	15.37	15.11	0.26	1.69	Khalil et al. (2020)
Wheat 1	39.1	14.15	13.84	0.31	2.19	Khalil et al. (2020)
Wheat 2	34.4	14.67	14.38	0.29	1.98	Perera et al. (2019)
Sorghum	39.5	15.60	15.32	0.28	1.79	Khalil et al. (2020)
Barley 1	41.2	13.43	13.10	0.33	2.46	Khalil et al. (2020)
Barley 2	45.5	13.67	13.39	0.28	2.05	Perera et al. (2019)

Δ AME = AME − AMEn.

**Table 8:** Composition and calculated analysis (%) of starter, grower, and finisher maize-soybean meal-based diets formulated using either apparent metabolizable energy (AME) or N-corrected AME (AMEn) values for maize and soybean meal—An example ^1^.

Item	Inclusion (%)					
	Starter		Grower		Finisher	
	AME	AMEn	AME	AMEn	AME	AMEn
Maize	56.63	53.40	60.84	57.86	64.89	62.16
Soybean meal 48%	38.48	39.00	33.75	34.22	29.02	29.46
Soybean oil	0.43	3.14	1.34	3.84	2.42	4.71
Dicalcium phosphate	1.660	1.660	1.485	1.490	1.310	1.315
Limestone	0.960	0.953	0.890	0.882	0.816	0.811
DL Methionine	0.354	0.359	0.310	0.315	0.278	0.282
L Lysine HCl	0.414	0.406	0.375	0.367	0.335	0.328
L Threonine	0.235	0.234	0.199	0.199	0.163	0.163
L Valine	0.090	0.091	0.072	0.073	0.054	0.055
Sodium bicarbonate	0.398	0.394	0.387	0.383	0.339	0.335
Sodium chloride	0.090	0.096	0.098	0.104	0.107	0.112
Vitamin premix	0.100	0.100	0.100	0.100	0.100	0.100
Mineral premix	0.100	0.100	0.100	0.100	0.100	0.100
Choline chloride 60%	0.065	0.066	0.066	0.067	0.067	0.069
**Calculated analysis**						
Apparent metabolisable energy (MJ/kg)	12.60	12.60	12.98	12.98	13.39	13.39
Crude protein	23.0	23.0	21.0	21.0	19.0	19.0
Starch	35.5	33.5	38.0	36.20	40.49	38.80
Crude fat	2.77	5.15	3.70	5.90	4.79	6.81
Crude fiber	2.93	2.88	2.82	2.77	2.70	2.66
Total calcium	0.96	0.96	0.87	0.87	0.78	0.78
Total phosphorus	0.70	0.70	0.64	0.64	0.59	0.59
Non phytate P	0.48	0.48	0.44	0.44	0.39	0.39
Phytate P	0.22	0.22	0.20	0.20	0.20	0.20
Chloride^-^	0.19	0.19	0.19	0.19	0.19	0.19
Sodium^+^	0.19	0.19	0.19	0.19	0.18	0.18
Potassium^+^	1.16	1.16	1.06	1.06	0.95	0.95
Choline (mg/kg)	1700	1700	1600	1600	1500	1500
Digestible threonine	0.86	0.86	0.77	0.77	0.68	0.68
Digestible valine	0.96	0.96	0.87	0.87	0.78	0.78
Digestible isoleucine	0.77	0.77	0.70	0.70	0.63	0.63
Digestible lysine	1.28	1.28	1.15	1.15	1.02	1.02
Digestible arginine	1.32	1.33	1.20	1.20	1.08	1.08
Digestible cysteine	0.31	0.30	0.40	0.40	0.37	0.37
Digestible methionine	0.64	0.65	0.47	0.47	0.43	0.43
Digestible methionine + cysteine	0.95	0.95	0.87	0.87	0.80	0.80
Energy contribution from maize (%)	63.6	58.9	66.3	62.0	68.5	64.5
Energy contribution from soybean meal (%)	33.9	30.9	28.9	26.3	24.1	21.9
Energy contribution from soybean oil (%)	1.2	9.0	3.7	10.7	6.5	12.7
Cost (NZ$ per tonne) ^2^	652	722	652	716	656	715

^1^ The following AME and AMEn values used for maize and soybean meal in the feed formulation: Maize, AME = 14.14 MJ/kg and AMEn = 13.90 MJ/kg; Soybean meal, AME = 11.10 MJ/kg and AMEn = 9.96 MJ/kg (average of 9 soybean meal samples from Table 3). ^2^ 1.00 NZ dollar = 0.72 US dollar (June 2021).

## Data Availability

All available data incorporated in the manuscript.
